# Commentary “A Crisis in Comparative Psychology: Where have all the Undergraduates Gone?” Collaborating with Behavior Analysts Could Avert a Crisis in Comparative Psychology

**DOI:** 10.3389/fpsyg.2015.01743

**Published:** 2015-11-13

**Authors:** Elizabeth G. E. Kyonka, Shrinidhi Subramaniam, Daniel Bell-Garrison, Matthew L. Eckard

**Affiliations:** Psychology, West Virginia UniversityMorgantown, WV, USA

**Keywords:** behavior analysis, comparative psychology, collaboration, recruitment, cross-species analysis

Abramson's crisis is that, due to lack of interest and opportunities, psychology undergraduates are not pursuing advanced study in comparative psychology at a rate sufficient to sustain it as a discipline. However, what Abramson has not considered is that strictly behavioral comparative research happens frequently in psychology departments under the label “behavior analysis.” This work is of high quality, frequently relates animal research to human behavior, and is broadly compatible with comparative cognition in spite of conflicting theoretical attitudes. Fostering communication and collaboration between behavior analysts, comparative cognition researchers, and traditional comparative psychologists is another way to avert the crisis of missing undergraduates in comparative psychology, both by tapping a group of undergraduates with established interests in behavior principles and by widening the base of active researchers in comparative psychology who can serve as potential supervisors to the next generation of comparative psychologists.

The experimental analysis of behavior is the basic science of behavior analysis (Morris et al., 2013), and its goal is to discover all of the variables that affect distributions of behavior (Skinner, 1966). It is essentially the study of fundamental behavioral principles that are applicable to all organisms (Baron and Perone, 1982; Palmer and Donahoe, 1991), making cross-species comparisons vital to the validation of phenomena that behavior analysts study. Zimmermann et al. (2015) identified 49 empirical studies published in the *Journal of the Experimental Analysis of Behavior* (JEAB) between 1958 and 2013 that reported results from multiple species. Many other articles report studies designed as explicit cross-species replications of previously published experiments (e.g., Macaskill and Hackenberg, 2013), and most research articles published in JEAB at least mention implications of the results for human behavior. Since 2013, JEAB has published experimental research comparing the behavior of different strains of mice (Minervini et al., 2015; Pope et al., 2015) and the behavior of pigeons with that of rats (Smethells and Reilly, 2015) and humans (Sweeney et al., 2014). These points illustrate that behavior analysts are publishing comparative psychology research in behavior-analytic journals. Comparative psychologists could increase the visibility of their discipline by considering non-comparative journals such as JEAB as outlets for their own work and by encouraging behavior analysts and others who do comparative research to publish in comparative psychology journals.

The goals of the experimental analysis of behavior are commensurate with those of comparative psychology. Citing Muckler (1963), Abramson identified a comparative analysis of human and animal behavior as “one of the major philosophical controversies in the intellectual tradition of the West,” and an integral component of comparative psychology. Similarly, the extent to which behavior principles generalize to human subjects is considered an indispensable empirical question in the experimental analysis of behavior (Baron et al., 1991a,b). Since the mid-twentieth century, the potential of operant learning theory to extend to a wide range of socially significant human behavior has been realized within the field of applied behavior analysis (Baer et al., 1968). Furthermore, there has been a recent push for so-called translational research within behavior analysis (Critchfield and Reed, 2009; and Mace and Critchfield, 2010; Critchfield, 2011). Special issues of JEAB (Mazur, 2010) and the *Journal of Applied Behavior Analysis* (Lerman, 2003) devoted to translational research constitute an exploration of connections between basic and applied counterparts and between nonhuman and human behavior.

Abramson acknowledged that comparative approaches are thriving outside psychology departments in areas such as integrative animal behavior and within psychology as comparative cognition. He questioned, however, whether “the study of behavior in comparative perspective, without reference to cognition,” is still a viable component of comparative psychology as it exists in psychology departments today. Without disputing the value of offering interested students a breadth of theoretical and philosophical approaches to comparative psychology, in our experience, comparative cognition is not as theoretically narrow as Abramson supposed: Zentall (2013) argued that the testable predictions of cognitive accounts of animal behavior should be of interest to behavior analysts. Several prominent comparative cognition researchers publish in JEAB, and there is enough interest in the subject among behavior analysts that JEAB editors plan to publish a special issue on comparative cognition in 2016 (A. L. Odum, personal communication, October 6, 2015).

Comparative research is being published and actively sought out by the flagship experimental behavior analysis journal with and without reference to cognition. Some of this work involves direct comparisons of the behavior of humans and other animals. We suspect that most behavior analysts, whether their own research is comparative or not, would be happy to collaborate with comparative psychologists of any theoretical orientation and attend comparative psychology conferences, but may not be aware that such opportunities exist or are not confident that they would be welcomed. Any initiative from comparative psychologists is likely to reap large rewards in the form of enthusiastic behavior analyst colleagues. For instance, our research group studies interval timing and determinants of choice in pigeons, rodents, and humans. Although we are behaviorists operating in a behavior analysis doctoral training program, we are interested in extending our work to new species and to situations beyond the operant-conditioning chamber. We would welcome the experience, ideas, and expertise that any potential comparative psychologist collaborators would provide.

## Author contributions

EK organized and wrote the commentary based on intellectual contributions from each author. SS contributed the portion on translational research. DB and ME provided input and conducted literature searches.

## Funding

This research was supported in part by West Virginia University Senate Research Grant R-14-10.

### Conflict of interest statement

The authors declare that the research was conducted in the absence of any commercial or financial relationships that could be construed as a potential conflict of interest.
